# Heat stress responses of plants during adaptation to high temperature environments: A review of recent advances and insights

**DOI:** 10.1080/15592324.2026.2735211

**Published:** 2026-09-20

**Authors:** Xinsong Wei, Huankai Zhang, Ruize Ma, Jie Zang, Yan Wang, Caiyu Yu

**Affiliations:** a College of Life Sciences, Zaozhuang University, Zaozhuang, People's Republic of China; b State Key Laboratory of Wheat Improvement, Shandong Laboratory of Advanced Agricultural Sciences in Weifang, Peking University Institute of Advanced Agricultural Sciences, Weifang, Shandong, People's Republic of China; c Linqu County Longshan New Material Industry Development Service Center, Linqu, Shandong, People's Republic of China

**Keywords:** Abiotic stresses, heat stress, regulatory networks, major crops, stem cells

## Abstract

Plants, as sessile organisms, continuously encounter diverse environmental challenges, often experiencing multiple abiotic stresses during their development and growth. Among these stress events, heat has emerged as an increasingly severe factor because of global warming and currently poses a major threat to plant development, crop productivity, and agricultural sustainability. As a heat stress response, plants have evolved multilayered sensing and signaling systems that perceive heat cues and activate downstream protective and adaptive programs. This review summarizes recent knowledge regarding heat stress responses of *Arabidopsis thaliana* and major crops, including maize, rice, and wheat. We first outline the morphological, physiological, and molecular consequences of heat stress and then examine current models of heat perception and downstream regulatory networks. Particular emphasis is placed on second messenger signaling, transcriptional control, epigenetic regulation, post-transcriptional regulation, and heat memory. We also highlight stem cell homeostasis as an emerging component of heat adaptation that links stress signaling with developmental stability. Finally, we evaluate current strategies for improving crop heat tolerance, particularly during the reproductive stage, and discuss priorities for future research and breeding in a warming climate.

## 1. Introduction

Increasing greenhouse gas emissions driven by human activity have markedly increased the global surface temperature. Consequently, heat stress has gained prominence as a critical environmental constraint that disrupts plant physiological and metabolic homeostasis and reduces the yield potential of staple crops, including rice, maize, and wheat.^1^ Together with its direct effects on crop performance, heat stress could have wider socioeconomic effects by reducing agricultural sustainability and threatening food security.^2^

Plants are sessile species that cannot avoid unfavorable thermal conditions. Therefore, they rely on complex and highly dynamic response systems, the outputs of which differ across species, genotypes, tissues, and plant growth stages.^3^ Therefore, clarifying how plants sense elevated temperatures, coordinate downstream molecular responses, and sustain growth under heat stress is critical to comprehensively understand fundamental plant biology and develop heat-resilient crop varieties.

Heat stress influences plants at multiple levels, ranging from visible morphological damage to metabolic disruption, transcriptional reprogramming, and developmental instability. Recent works have reported substantial advances in discovering the regulatory networks underlying plant thermotolerance. However, several challenges remain. First, numerous current models are based predominantly on *Arabidopsis*, and their relevance to crop species remains partially resolved. Second, the classical heat stress response pathway, centered on heat shock proteins (HSPs) and heat shock factors (HSFs), has been extensively characterized.

However, increasing evidence indicates that complementary regulatory mechanisms, including chromatin remodeling, post-transcriptional RNA regulation, and meristem homeostasis, are vital for thermotolerance. This review first summarizes the major impacts of heat stress on plants, then discusses the mechanisms through which heat signals can be perceived and translated into downstream responses, and finally examines how these mechanisms may inform strategies to augment crop heat tolerance, particularly at the reproductive stage.

## 2. Effects of heat stress on plants

Heat stress describes the adverse effects that arise when environmental temperatures are greater than the range required for optimal plant growth.^4^ Under current climatic conditions, heat stress has emerged as a crucial abiotic constraint that restricts plant development, destabilizes agroecosystems, and threatens food security.^5^ Its impact spans multiple biological scales and extends beyond cellular damage. In general, three interrelated aspects can be used to explain how plants react to heat stress: structural and morphological damage, reprogramming of molecular pathways, and physiological and metabolic disruption.

### 2.1. Effects of heat stress on plant morphology

Compared to ideal growth conditions, high temperatures affect mostly all stages of plant development and typically reduce growth rate and growth-related characteristics, for example, stem diameter, plant height, and leaf area. Heat stress inhibits cell elongation and division during seed germination and the establishment of seedling, thereby reducing seedling emergence and early vigor. During the vegetative and reproductive development of the plant, long-term heat exposure can cause abortion of the reproductive organ, decreased fertility, aberrant female and male gametophyte growth, deformity of fruits, and a smaller grain size.^6^ ^,^ ^7^ These effects often result in lower yields at maturity and generate plant products with inferior quality.^8^ ^,^ ^9^

### 2.2. Effects of heat stress on plant physiological and biochemical characteristics

#### 2.2.1. Osmotic adjustment and oxidative stress

Plants experience significant fluctuations in metabolite abundance and enzyme activity during heat stress response. The majority of these changes are commonly used as physiological markers of stress tolerance. Malondialdehyde (MDA), hydrogen peroxide (H_2_O_2_), proline (Pro), and glutathione (GSH) are frequently examined metabolites, while antioxidant enzymes are the major enzymatic markers.^10^

Heat stress typically increases the activity of antioxidant enzymes while promoting the accumulation of Pro, MDA, GSH, and peroxide. Pro contributes to signaling, osmotic correction, and cellular defense.^11^ MDA is used as a measure of damage and peroxidation of membrane lipids.^12^ ^,^ ^13^ Reduced GSH has a key role in protecting cells from damage due to oxidative stress.^14^ Furthermore, because high temperatures disrupt chloroplast and mitochondrial functions, there is excessive accumulation of reactive oxygen species (ROS), such as H2O2, superoxide, and hydroxyl radicals.^15^ To mitigate this oxidative burden, the cells activate antioxidant enzymes such as glutathione peroxidase (GSH-Px), catalase (CAT), and superoxide dismutase (SOD) to minimize damage by eliminating ROS. ROS are not simply toxic byproducts; rather, they act as signaling molecules linking cellular damage with stress signaling and transcriptional reprogramming.

#### 2.2.2. Respiration

Respiration is an enzyme-mediated process through which organic compounds are oxidized to release energy required for growth and maintenance. Short-term exposure to elevated temperatures often enhances respiration and may temporarily support the synthesis of protective metabolites, protein turnover, and detoxification of stress-associated compounds. Under prolonged heat stress, sustained respiratory demand can quickly deplete carbon reserves and disrupt the activity of plant respiratory system-associated enzymes, thereby constraining growth and stress resilience. Heat stress may also reduce the efficiency of carboxylation by Rubisco while promoting photorespiration. Although photorespiration can contribute to short-term stress acclimation, its prolonged elevation may incur metabolic cost and further aggravate nitrogen-related imbalance.^16^

#### 2.2.3. Photosynthesis

Among plant physiological processes, photosynthesis is specifically vulnerable to impairment by heat stress.^17^ During a brief period of heat exposure, a decline in the photosynthetic rate is usually driven mainly by stomatal limitations,^18^ whereas under prolonged stress, impairment increasingly arises from non-stomatal constraints.^19^ Under sustained high temperatures, the loss of photosynthetic efficiency is largely associated with Rubisco inactivation and structural damage to photosystem II (PSII).^20^ Heat stress may further weaken the antioxidant defense mechanisms of the chloroplast and disrupt the integrity of chloroplasts and thylakoid membranes. Thus, photosynthesis is affected not only as an immediate target of thermal injury but also as a central process underlying subsequent growth suppression and yield reduction.

### 2.3. Effects of heat stress on plants at the molecular level

Heat stress induces a wider reorganization of the regulatory system at the molecular level, including changes in nucleosome occupancy, reprogramming of heat-responsive gene expression, induction of heat shock proteins coupled with related regulatory components, and chromatin remodeling. Rather than acting independently, these molecular processes function synergistically in tandem with physiological modifications and developmental regulation to determine whether plants incur damage, acclimatize to the environment, or recover from the injury.

### 2.4. Effects of heat stress on the reproductive stage

**Figure 1.**
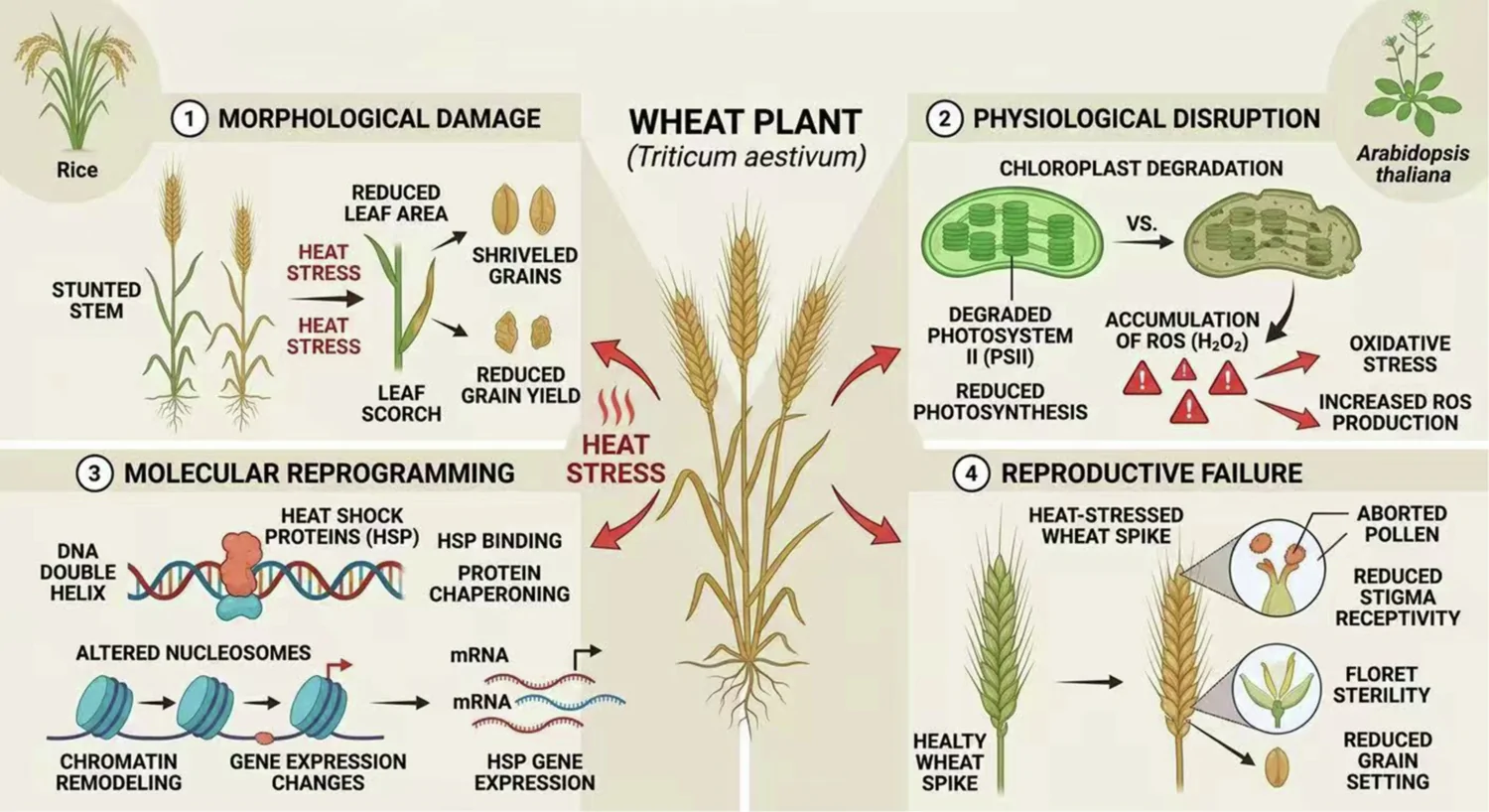
Schematic illustration of heat‑stress responses in wheat (Triticum aestivum). Heat stress triggers four major effects in wheat: (1) morphological damage (stunted stem, leaf scorch, shrveled grains and reduced yield); (2) physiological disruption including chloroplast degradation, photosystem II (PSII) impairment, inhibited photosynthesis, and accumulation of reactive oxygen species (ROS, H₂O₂) leading to oxidative stress; (3) molecular reprogramming via chromatin‑remodeling‑driven gene expression changes and heat‑shock protein (HSP)‑mediated protein chaperoning; (4) reproductive failure with aborted pollen, reduced stigma receptivity, floret sterility and poor grain setting. Rice and Arabidopsis thaliana are included as reference plant species.

One of the most thermosensitive phases in a plant’s life cycle is the reproductive stage, and the damage sustained during this phase often directly impacts fruit set and final production. Elevated temperatures disrupt normal floral development by causing abnormalities in pollen mother cell meiosis, lowering pollen viability, impairing anther dehiscence, and reducing stigma receptivity, thereby compromising pollination and fertilization. Heat stress not only impairs embryo development and grain filling but also decreases the activity of enzymes participating in starch synthesis, which ultimately causes grain abortion, low grain weight, and poor quality of grains.^21^ Moreover, high ROS accumulation and disturbance of hormone homeostasis exacerbate reproductive failure. From an applied perspective, this stage is particularly important because it directly links cellular heat responses to agricultural productivity (Figure 1).

## 3. How plants perceive heat stress

The plant mechanism mediating the conversion of variations in intracellular and extracellular temperatures to physiological responses is termed “heat perception.” According to current evidence, plant heat sensing can be best understood as a distributed and multilayered process rather than a single linear channel. Several cellular components appear to be involved in decoding elevated temperatures through changes in physical characteristics, molecular interactions, and metabolic activity.^22–24^ Overall, heat exposure initially induces structural alterations in cellular components, for example, in the plasma membrane and cell wall, thereby triggering second messenger production. These second messengers include Ca^2+^, ROS, and lipid-derived signals. The amplification and propagation of these signals through processes such as subcellular relocalization and protein phase separation eventually activate the heat stress response (HSR) pathway. Therefore, heat perception is more correctly viewed as a multilayered input system that converges on downstream regulatory networks.

### 3.1. Heat perception mediated by cellular structural changes

The cell wall is considered one of the initial sites involved in detecting high temperatures. Heat stress increases the activity of pectin methylesterase (PME) on cell wall pectin, which promotes proton release via demethylation. This leads to a decrease in apoplastic pH, activation of endopolygalacturonase, loosening of the cell wall, and release of Ca^2+^ chelated in the extracellular matrix.^25^ Calcium-permeable channels localized in the plasma membrane allow the released Ca^2+^ to enter the cytoplasm, subsequently activating downstream HSR pathways.^25^ ^,^ ^26^ This cell wall-based thermosensing mechanism is not universal across species; for example, maize exhibits stronger PME-mediated heat responses compared to rice, suggesting lineage-specific tuning of cell wall heat sensing.^26^ Thus, these findings indicate that structural perturbations may function as a critical inducer of heat signaling.^27^

### 3.2. Early heat signal transduction mediated by second messengers

When structural changes caused by heat stress commence signaling, the cells rapidly produce a number of second messengers. Notably, the three major signaling modules are Ca^2+^, ROS, and lipid signals. Furthermore, nitric oxide (NO) signaling, specifically nitrate reductase (NR)-mediated NO signaling, has a critical contribution to heat tolerance.^28^ Instead of functioning independently, these messengers form a coordinated signaling network that collectively regulates the early response to heat stress.^29^

#### 3.2.1. Calcium signaling pathway

Ca^2+^ is one of the initial signals generated by plants in their response to heat stress. Elevated temperatures facilitate Ca^2+^ influx by activating two Ca^2+^-permeable channels localized in the plasma membrane: annexin 1 (ANN1) and cyclic nucleotide-gated channels (CNGCs).^30^ While ANN1 activation is dependent on H_2_O_2_-mediated ROS signaling, the activation of CNGCs is stimulated by cyclic adenosine monophosphate or cyclic guanosine monophosphate.^31^ The subsequent rise in the Ca^2+^ level in the cytosol initiates the expression of respiratory burst oxidase homolog D (RBOHD), increasing ROS generation and creating a Ca^2+^-ROS positive feedback loop.^32^ This reciprocal amplification could be a distinguishing characteristic of early heat signaling.

#### 3.2.2. Lipid signaling pathway

Another essential element of plant heat response is lipid signaling. Phospholipase D (PLD) and the phospholipase C/diacylglycerol kinase (PLC/DGK) pathway are rapidly stimulated by heat stress, resulting in the synthesis of signaling lipids, such as phosphatidic acid (PA).^33^ PA not only affects the integrity of the plasma membrane but also interacts with downstream target proteins, altering their activities.^34^ Furthermore, inositol trisphosphate is released through PLC-mediated hydrolysis of phosphatidylinositol, which enables Ca^2+^ release from intracellular stores and further enhances Ca^2+^ signaling. These findings suggest that membrane lipid remodeling has a dual role of serving as an active signaling output and functioning as a biophysical result of heat exposure.

#### 3.2.3. ROS signaling

During heat stress, RBOHD activation accelerates ROS generation. In the plant heat response, ROS serve as signaling cues that alter heat-responsive gene expression in addition to operating as reactive intermediates of redox metabolism. ROS can enhance Ca^2+^ influx and augment the broader signaling network by activating Ca^2+^ channels in the plasma membrane. Thus, ROS should be regarded as having a dual function in heat responses: they promote cellular injury and act as an essential mediator of propagating stress signals.^35^

### 3.3. Heat signal transmission mediated by protein dynamics

Plants are equipped with other heat sensors in addition to second messenger cascades or structural disturbances. Accumulating data suggest that protein dynamics contribute critically to heat signal transmission. Specifically heat-induced phase separation and subcellular localization shift probably modulate the pathways through which thermal stimuli are relayed, assimilated, and interpreted.

#### 3.3.1. Signal regulation mediated by protein phase separation

Liquid–liquid phase separation (LLPS) has been recently recognized as a crucial mechanism in plant heat sensing and stress adaptation. Under heat stress, selected proteins undergo phase separation and assemble into stress granules (SGs), thereby regulating RNA stability and translation. Glycine-rich RNA-binding proteins RBGD2 and RBGD4 can form heat-induced SGs through LLPS, associate with heat-responsive mRNAs, and promote their maintenance, thereby facilitating downstream gene expression.^36^ ^,^ ^37^

Similarly, in *Arabidopsis*, an intrinsically disordered protein FUST1 can sense elevated temperatures through phase separation of its structural domain. This aggregation process is necessary to assemble heat SGs and facilitate basic heat tolerance.^38^ An identical phenomenon was noted for acetylation lowers binding affinity (ALBA) proteins. Under elevated temperatures, these proteins undergo phase separation and generate SGs, along with processing bodies that associate with heat shock factor mRNAs and support mRNA stability.^39^ Although the physiological significance of these condensates has not yet been fully established, current evidence supports the notion that phase separation provides a rapid and reversible mechanism for reconfiguring gene expression during heat stress.

#### 3.3.2. Changes in protein subcellular localization

**Figure 2.**
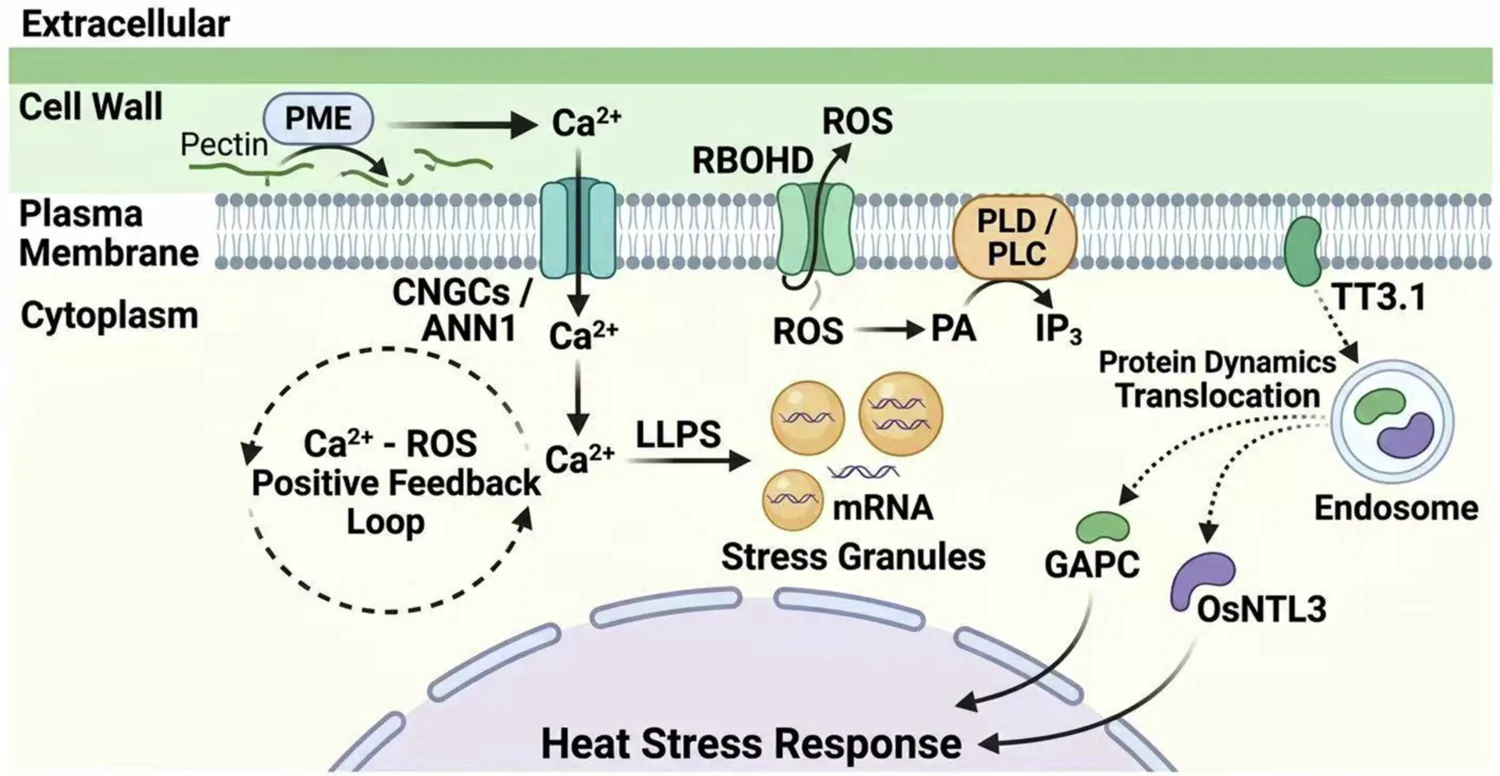
Schematic model of early‑heat‑stress signaling events in plant cells. Upon heat stress, pectin methylesterase (PME) modifies cell‑wall pectin to trigger cytosolic Ca^2^⁺ influx via cyclic nucleotide‑gated channels (CNGCs) and annexin1 (ANN1). Ca^2^⁺ and reactive oxygen species (ROS) form a positive‑feedback loop. Ca^2^⁺‑driven liquid‑liquid phase separation (LLPS) promotes stress‑granule assembly containing mRNA. Plasma‑membrane‑localized respiratory‑burst‑oxidase homolog D (RBOHD) produces ROS. Phospholipase D/C (PLD/PLC) generates phosphatidic acid (PA) and inositol trisphosphate (IP₃). Endosome‑mediated protein translocation of TT3.1 releases OsNTL3 and GAPC, which collectively activate downstream heat‑stress‑response pathways.

Translocation of proteins between cellular compartments is induced by high temperatures, thereby initiating downstream signaling and modification of interaction networks. Heat causes the cytoplasmic-to-nucleus translocation of glyceraldehyde-3-phosphate dehydrogenase, where it interacts with the transcription factor (TF) nuclear factor Y subunit C10 to promote downstream heat responses.^40^ This process depends on PA and PLDδ.^41^ Heat treatment also promotes the membrane-to-nucleus translocation of the NAC TF OsNTL3 that activates downstream heat-responsive pathways.^42^ Additionally, inter-organelle communication may participate in heat sensing. Thermos-tolerance 3.1 (TT3.1), which localizes to the plasma membrane, has been proposed to respond to extracellular heat cues through relocation to endosomes, thereby contributing to intracellular heat signal decoding.^43^ These observations indicate that heat sensing involves the dynamic redistribution of proteins, rather than a single receptor-like event (Figure 2).

## 4. How plants respond to heat stress

Following heat perception, plants rapidly engage a multilayered molecular regulatory system that limits cellular injury while preserving growth and developmental homeostasis.^44^ Broadly, plant responses to elevated temperatures involve the activation of transcriptional regulatory networks, further modulation through post-transcriptional and epigenetic control, and the establishment and resetting of heat memory. These processes are not independent modules. In contrast, they constitute an integrated regulatory framework in which early signaling, transcriptional activation, chromatin status, RNA metabolism, and proteostasis are closely interlinked.

### 4.1. Core role of transcriptional regulatory networks in heat responses

#### 4.1.1. HSF–HSP pathway

Heat shock factors (HSFs) are the best-characterized TFs involved in plant heat responses. They regulate downstream gene expression by binding to heat shock elements (HSEs; nGAAn motifs).^45^ In maize, *ZmHSF12-1* and *ZmHSF12-2* overexpression can substantially increase plant heat tolerance, and the co-expression of these two genes can compensate for the growth defects caused by high *ZmHSF12-2* expression.^46^ In tomato, overexpression of *HSFA1* enhances heat tolerance, whereas co-suppressed lines exhibit increased heat sensitivity and impaired HSP synthesis.^33^ In *Arabidopsis*, *HsfA1a*, *HsfA1b*, and *HsfA1d* positively regulate heat-responsive gene expression by binding to the HSE sequence in the *DREB2A* promoter, and triple mutants exhibit severe defects in heat tolerance.^47^ HSFA2 acts downstream of HSFA1 proteins, and HSFA2 loss markedly compromises acquired thermotolerance. HSFA2 also supports the sustained expression of heat-responsive genes through positive feedback regulation.^48^ In barley, *HvHSFA2* and *HvHSFA3* regulate heat stress memory through a differentiation-dependent mechanism.^49^

The functions of HSF family members are broadly conserved across species.^50^ In soybean, *GmHSFA1* augments heat tolerance by activating *GmHSP70* and *GmHSP22* expression.^51^ The expression of *HSFA9* and *HSP70* in tomato is enhanced in the auxin signaling inhibitor (SIIAA9) mutant, which improves resistance to high-temperature stress.^52^ In wheat, *TaHSFA6f* improves heat tolerance by upregulating *TaHSP90.1-A1* and *TaGAAP.* ^53^ *TaHSF3*, which shows a high expression in spikes and is induced by multiple stress factors, including low temperature and drought, enhances heat tolerance by activating *HSP70* expression.^54^ In rice, similar conserved regulatory patterns have also been reported. OsHSFA4d activates its expression by binding to the heat shock element in HSP101 promoter. In addition, OsHSFA4d is phosphorylated by OsCDPK24 and OsCDPK28 to enhance its DNA-binding ability, ultimately significantly improving plant heat tolerance.^55^ In addition, *HSFA4* activates *APX1* expression under oxidative and heat stress, whereas HSFA5 represses HSFA4 activity through the formation of dimers.^56^ ^,^ ^57^

Collectively, these findings establish the HSF-HSP pathway as the central regulatory axis of plant thermotolerance. This pathway should not be regarded as an isolated module because its activity is tightly integrated with ROS, hormones, RNA, and chromatin-based regulatory processes.

Notably, the HSF hierarchy model derived from Arabidopsis is not fully conserved in crops. For instance, wheat has a more expanded HSF family with functional subfunctionalization, and some rice HSF members exhibit stress-specific expression patterns not observed in Arabidopsis. These lineage-specific differences are important considerations for translating model organism findings to crop improvement.^58–60^

#### 4.1.2. Coordinated regulation by DREB and other TFs

Beyond HSFs, DREB TFs also function as the major regulators of plant heat responses. As an AP2/ERF family member, *DREB2A* is rapidly induced by heat stress and promotes thermotolerance by regulating dehydration- and heat-responsive genes.^61^ ^,^ ^62^ *DREB2C* additionally contributes to heat tolerance by activating *HSFA3* expression.^63^ Identical roles are noted in crops, where maize *DREB2A*, rice *OsDREB2B*, and lily *LlDREB1G* are related to enhanced heat tolerance.^64–67^

Heat responses are also influenced by other TF families, including MYB, NAC, and WRKY. *WRKY39* enhances thermotolerance by coordinating salicylic acid and jasmonic acid (JA) signaling.^68^ *WRKY25*, *WRKY26*, and *WRKY33* show functional redundancy and jointly mediate ethylene-dependent responses triggered by high temperatures.^69^ In rice, *WRKY11* overexpression can remarkably enhance tolerance to both drought and heat.^70^ Among MYB factors, *OsMYB55* promotes heat tolerance during vegetative growth by regulating amino acid metabolism-related genes,^71^ *MYB68* enhances thermotolerance by enhancing root lignin accumulation,^72^ and wheat *MYB80* is linked with abscisic acid (ABA) level and anthocyanin biosynthesis.^73^ NAC TFs, including *JUB1*, *NTL3*, *SNAC3*, and *TaNAC2L*, also regulate heat stress responses by maintaining reactive ROS homeostasis, regulating the unfolded protein response (UPR), and activating genes associated with acquired thermotolerance.^74–76^

These TFs should not be viewed merely as additional entries in the catalog of heat-responsive regulators. These findings indicate that plant thermotolerance is coordinated through partially overlapping subnetworks that link canonical HSF-centered responses with broader developmental, hormonal, and redox regulation.

#### 4.1.3. Regulatory mechanisms controlling key TF Activity

Plant responses to elevated temperatures rely on TF abundance and stringent control via post-translational modification and protein interactions. As a representative example, both phosphorylation and protein–protein interactions control the activity of HSFA1.^77^ In *Arabidopsis*, *HSFA1* is phosphorylated by CBK3 and CDKA-related kinase, which modulates its ability to bind DNA.^78^ ^,^ ^79^ Wheat also shows identical protein–protein interactions and phosphorylation regulatory mechanisms. Heat stress can diminish *TaSERL2* kinase-mediated modification of *TaBZR2* through phosphorylation, thereby impairing its inhibitory effect on protein degradation. This strategy improves heat tolerance by increasing the expression levels of downstream heat-responsive genes.^80^ *PP7*, an *HSFA1*-interacting phosphatase, enhances heat tolerance by triggering *HSP101* and *HSP70* expression.^81^ Members of the *HSP90*, *HSP70*, and *HSC70* families negative regulate *HSFA1* under nonstress conditions by repressing its nuclear localization or DNA-binding activity.^82–84^ MYB TFs can regulate both HSF pathway and plant antioxidant system. ZmMYB104 binds to *ZmCAT2* promoter in maize to initiate its expression. This regulatory step minimizes oxidative damage by enhancing ROS scavenging at high temperatures, eventually increasing plant heat tolerance.^85^

*DREB2A* is also subject to stringent regulations. At the transcriptional level, *HSFA1s*, *MBF1c*, and *JUB1* stimulate *DREB2A* expression under heat stress, while *GRF7* represses its expression under normal temperatures.^86^, ^87^ At the protein level, DREB2A’s *N*-terminal negative regulatory domain controls its proteolytic degradation mediated by the 26S proteasome pathway. This regulation mechanism involves E3 ligases, including *DRIP1/2* and *BPM-CUL3*.^88–92^ Furthermore, *DREB2A*’s stability and transcriptional activity are enhanced by SUMOylation due to high temperatures.^93^ ^,^ ^94^ These multilayered regulatory mechanisms illustrate the overall pathway of the response of plants to heat stress: rapid activation combined with stringent control to avoid unnecessary growth penalties or maladaptive signaling.

### 4.2. Role of epigenetic and post-transcriptional regulation in heat responses

In addition to TF-dependent rapid activation, plant heat responses are also modulated by post-transcriptional regulation and epigenetic control. These mechanisms, which are specifically crucial for stress memory and developmental adaptation, refine the amplitude, persistence, and timing of heat-responsive gene expression.

#### 4.2.1. Epigenetic regulation

Epigenetic regulation is a crucial process through which plants modulate heat responses and establish heat memory. Through chromatin remodeling and histone modification, the transcriptional competence of heat-responsive genes can be adjusted without altering the underlying DNA sequence. H3K4me3 is widely regarded as an important molecular marker associated with heat memory. In *Arabidopsis*, heat-responsive gene loci, such as *HSP18.2*, *HSP21*, and *HSP22.0*, maintain elevated H3K4me3 levels after recovery from heat stress. This modification facilitates faster reactivation during subsequent heat exposure.^95^ HSFA2 also contributes to the enrichment of H3K4me2/me3 at memory-associated loci and has a key role in sustaining heat-responsive gene expression.^95^ ^,^ ^96^

Together with histone methylation, nucleosome remodeling and histone acetylation regulate heat tolerance. In *Arabidopsis*, *ASF1A* and *ASF1B* activate genes such as *HSFA2* by promoting H3K56ac and reducing nucleosome occupancy at target loci, facilitating transcriptional activation.^97^ *FGT1* can cooperate with chromatin remodeling factors, including *BRM* and *CHR11/17*, to preserve an open chromatin state at heat memory loci and maintain their expression.^98^ The abovementioned observations suggest that, besides acting as a downstream consequence of heat responses, chromatin status functions as an active regulatory layer determining the duration and recall capacity of thermotolerance. In crops, epigenetic heat memory has important practical applications: heat priming of seeds or seedlings can induce lasting epigenetic changes that improve heat tolerance later in development, providing a cost-effective agronomic strategy for field production.^99^

#### 4.2.2. Post-transcriptional regulation

Plants also modulate the expression of heat-responsive genes through post-transcriptional mechanisms, particularly those involving alternative splicing and small RNAs. The central components of this regulatory layer are microRNAs (miRNAs). In *Arabidopsis*, *miR156* is induced under heat conditions and extends heat memory by downregulating *SPL* TFs, thereby coordinating the balance between developmental progression and stress responses.^100^ The *miR398* gene promotes ROS accumulation by repressing the ROS-scavenging genes *CSD1*, *CSD2*, and *CCS*, which further activates the HSFA1-dependent heat response pathway and establishes a positive feedback loop.^101–103^ In wheat, TaIRE1-mediated regulation of *miR172* precursor is closely related to heat stress responses.^104^

Together with the regulation of intracellular homeostasis and plant growth, miRNAs can serve as a hub to connect the cross-defensive pathways of heat tolerance and disease resistance. In rice, *miR444b.2* suppresses *HsfA1* expression by targeting and cleaving its mRNA. Under high-temperature stress, this miRNA is downregulated, thereby activating the downstream JA biosynthesis pathway and enhancing both heat tolerance and rice blast resistance.^105^

Heat-induced *TAS1* also contributes to thermotolerance regulation. By targeting *HTT1/2*, the function of the Hsp70-related complex is influenced, thereby participating in the *HSFA1a*-dependent heat tolerance response.^106^ ^,^ ^107^ In addition, tocopherol can enhance miRNA biogenesis by promoting *PAP* accumulation and inhibiting *XRN*-mediated degradation of pri-miRNAs. This can improve heat tolerance.^108^ Similarly, alternative splicing is a pivotal component of post-transcriptional regulation at high temperatures. Following heat stress, certain heat-responsive genes retain particular splicing patterns; this feature affects their efficiency of expression and functional output when the plant is again exposed to heat. This mechanism is strongly related to transcriptional heat memory.^109^

Epigenetic and post-transcriptional regulation serve as a pivotal fine-tuning layer in plant heat responses. The former system potentially alters the chromatin state to modulate gene expression, whereas the latter pathway largely operates through RNA processing, stability, and translational efficiency. The combination of these regulatory processes highlights a key aspect of thermotolerance—this ability relies not only on the type of activated genes but also on their activation duration, expression strength, and developmental context of their expression.

### 4.3. Heat memory and its resetting mechanisms

Under recurrent high-temperature conditions, plants establish heat memory, enabling quicker and robust protective responses to subsequent heat exposure.^110^ This memory includes short-term somatic memory and transgenerational memory.

Transposons appear to contribute in a distinctive way to transgenerational heat memory. Studies in *Arabidopsis* have revealed an interaction between HSP70-3 (a heat shock protein) and SGS3 (a plant-specific silencing suppressor). This interaction has a crucial regulatory role in creating and sustaining transgenerational heat memory by modulating downstream heat-responsive genes and transposable element activity.^111^

In addition, in *Arabidopsis*, the retrotransposon *ONSEN* contains HSEs and is activated by *HSFA1* and *HSFA2* at high temperatures.^112^ In mutants impaired in *siRNA* biogenesis, *ONSEN* can produce additional copies after heat stress and transpose in the following generation, which can alter the expression of neighboring genes and create new heat-responsive regulatory relationships.^98^ *HSFA2* forms a positive feedback loop with the H3K27me3 demethylase *REF6*, while this regulatory configuration has been proposed to influence flowering time and immune responses in progeny.^66^ ^,^ ^67^ At room temperature, the chromatin remodeling factors *DDM1* and *MOM1* are involved in restoring transposon silencing and maintaining genomic stability.^113^ These findings suggest that in some contexts, heat memory may extend beyond transient acclimation, although the generality and extent of transgenerational inheritance remain to be clarified.

In addition to memory establishment, plants also need to reset their response state after stress recovery to resume their normal growth. This process is mediated by autophagy. For example, heat-inducible expression of ZmATG8c in maize improves heat tolerance during both reproductive and vegetative growth stages.^114^ Heat pretreatment can induce sustained autophagy. ATG8 can bind proteins such as HSP101, HSP17.6, and HSP90 and promote their autophagosome-based degradation. Besides, ATG8 stability is precisely controlled by the Arg/N-degron pathway. Collectively, these regulatory processes enable plants to rapidly remove damaged proteins, restore cellular homeostasis under heat stress,^115^ and facilitate the transition from the stress state back to cellular homeostasis.^116^ Following autophagy blockade, HSPs accumulate excessively, heat memory becomes abnormally prolonged, and resource reallocation and post-stress recovery are impaired.^116^

**Figure 3.**
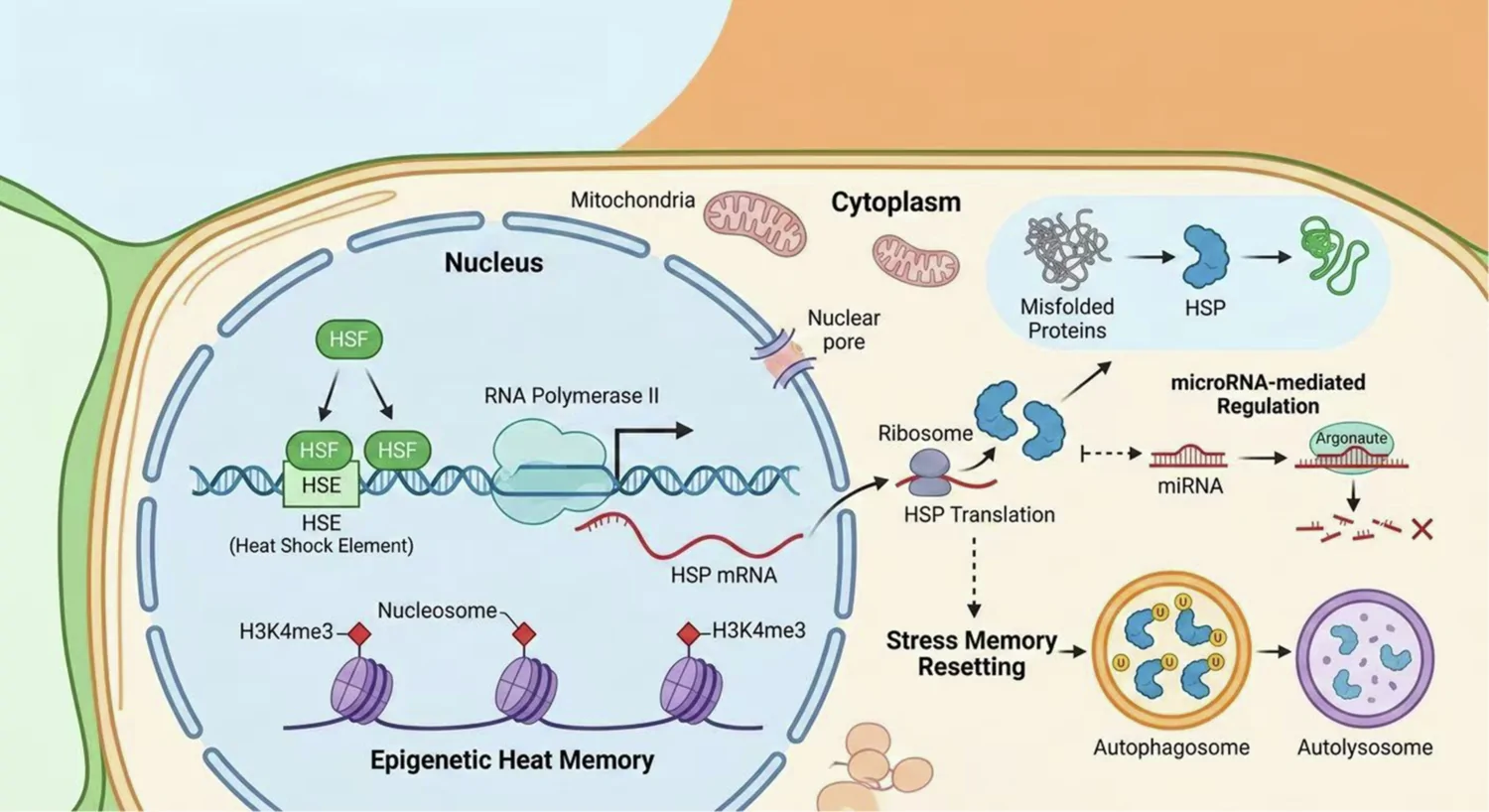
Schematic model of transcriptional, epigenetic and post‑transcriptional regulation under plant heat stress. In the nucleus, heat‑shock factors (HSFs) bind to heat‑shock elements (HSEs) and recruit RNA polymerase II to drive transcription of heat‑shock protein (HSP) genes. Histone modification H3K4me3 participates in epigenetic heat‑memory formation. Exported HSP mRNAs are translated into HSPs in the cytoplasm; HSPs function to refold misfolded proteins. MicroRNA (miRNA)‑Argonaute complex mediates post‑transcriptional repression. Autophagy‑dependent stress‑memory resetting degrades target substrates via autophagosome‑autolysosome pathways.

Thus, both the establishment and resetting of heat memories are essential. This balance is conceptually important because thermotolerance does not simply depend on maintaining stress responses for as long as possible; efficient recovery is equally important for sustained growth (Figure 3).

## 5. Role of stem cell homeostasis in plant heat tolerance

Beyond the classical framework of heat perception and response, recent studies have suggested that maintaining stem cell homeostasis in meristems is a critical component of plant heat tolerance. The shoot apical meristem (SAM) and root apical meristem (RAM) harbor stem cell populations exhibiting the characteristic of continuous division and differentiation; therefore, preservation of their homeostasis is pivotal to manage growth and formation of organs under heat stress. This perspective is significant because it established a link between developmental continuity and stress signaling. Although canonical heat-response pathways primarily clarify how cells tolerate heat exposure, stem cell homeostasis demonstrates how plants retain their growth potential and developmental stability both under and after stress.

### 5.1. Regulatory mechanisms of stem cell homeostasis under heat stress

Plant growth and development are considerably impacted by the ongoing activity of SAM and RAM stem cell populations.^117–119^ Under heat stress, these cells have to sense fluctuations in temperature and maintain a dynamic equilibrium between preserving proliferative ability and restricting cellular damage. WUSCHEL (WUS), the primary stem cell homeostasis regulator, not only helps plant tolerate heat^120^ but also ensures that maintenance of stem cells, formation of organs, and reproductive transition are well balanced.^121^

Under normal conditions, MYB3R-like proteins interact with rId2 methyltransferase to maintain the stability of WUS mRNA by controlling its 5′ cap structure, thereby sustaining WUS expression and promoting consistent stem cell functioning.^122^ The MYB3R-like/rId2 module is inhibited by heat stress, which lowers the stability of WUS mRNA. This response may help restrict misfolded protein accumulation at high temperatures, thereby partially alleviating proteostasis stress in meristematic cells.^122^ Thus, the dynamic regulation of WUS reflects a balancing mechanism between developmental maintenance and damage limitation under heat stress. Similarly, heat-induced ROS signaling in tomato delays the maturation of apical meristem. This mechanism provides an additional regulatory step to maintain stem cell homeostasis and heat tolerance during plant development.^123^

There is a close relationship between stem cell tolerance to elevated temperatures and protein quality control. By activating the unfolded protein response (UPR) and related protein folding and repair pathways, plants can strengthen meristematic cells’ capacity to detect, repair, and remove misfolded proteins.^124^ ^,^ ^125^ Accordingly, the maintenance of stem cell homeostasis depends not only on developmental regulatory circuits but also on tight coupling with intracellular proteostasis under heat stress. This relationship provides an important conceptual bridge between meristem maintenance and a broader heat-response network. Although stem cell homeostasis cannot replace canonical thermotolerance pathways, it possibly integrates developmental regulation with proteostasis and recovery capacity.

### 5.2. Potential of stem cell homeostasis-related mechanisms for improving heat tolerance

Stem cell homeostasis-related pathways may provide new avenues to enhance plant heat tolerance. Under high temperatures, the regulation of the MYB3R-like/rId2-WUS module could improve meristem maintenance, thereby facilitating the continuity of plant growth under heat stress. Similarly, meristems’ capacity to cope with protein damage could be strengthened by activating WUS-associated UPR components, including protein folding factors such as bZIP28 and BiP2.^124^ ^,^ ^125^

Beyond offering local protection, meristems may also be involved in systemic signaling of heat tolerance. After high temperature perception, the SAM generates signaling molecules, such as S-nitrosoglutathione, and transmits heat signals through the vascular system, leading to a systemic heat response network formation.^126^ Moreover, stem cells may help preserve genome stability by maintaining epigenetic silencing of heat-responsive transposons through mechanisms such as DNA methylation. These findings indicate that the stem cell homeostasis process is a critical mechanism that links local stress defense, developmental continuity, and whole-plant coordination. Therefore, regulatory factors such as rId2 and WUS represent promising candidate targets for future genetic engineering modifications aimed at improving heat tolerance.

**Figure 4.**
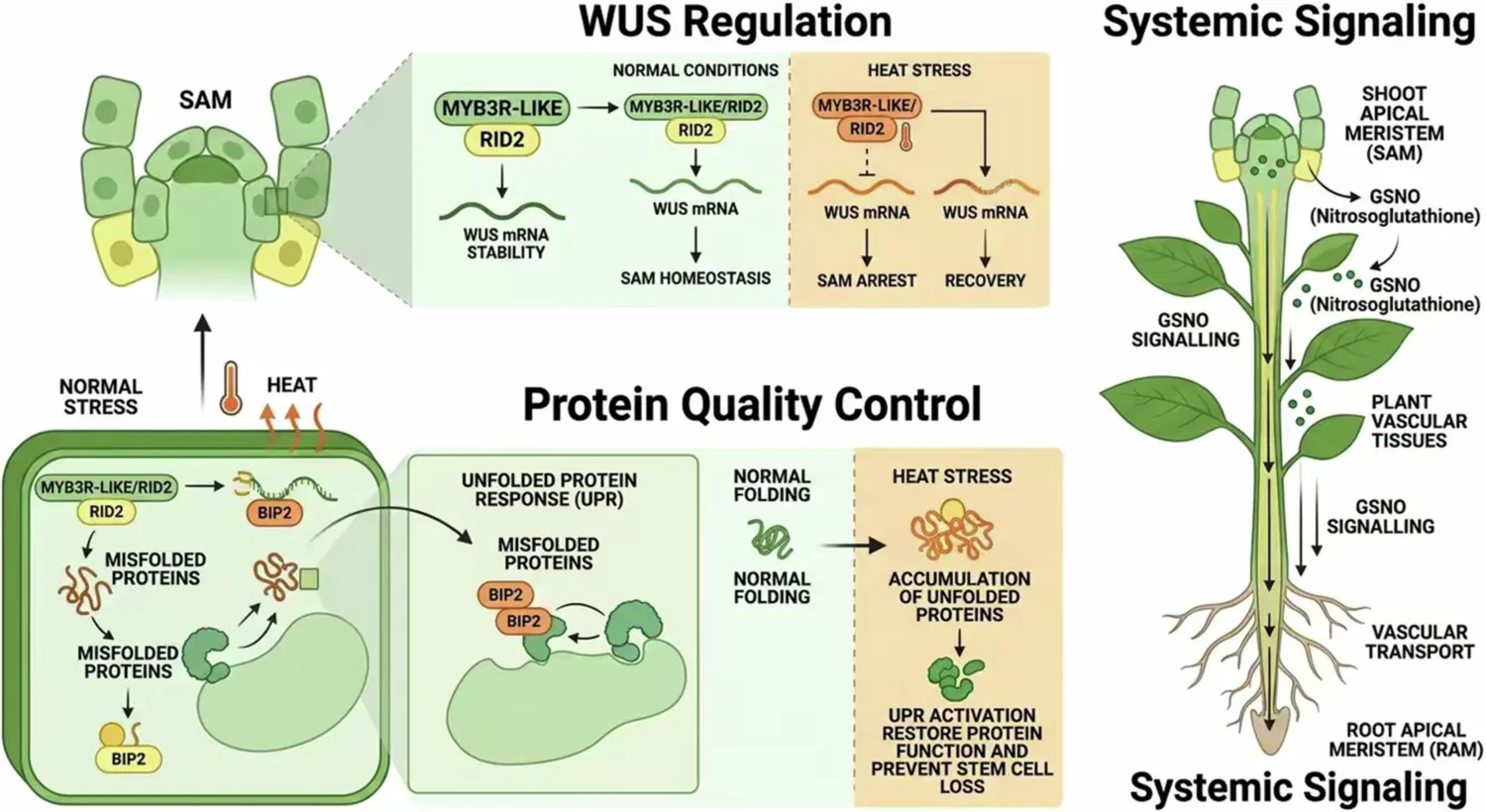
Schematic diagram illustrating heat‑stress‑related regulatory modules in plant meristems. Three key regulatory modules are presented: WUS regulation, protein quality control, and systemic signaling. Under normal conditions, MYB3R‑LIKE/rId2 maintains WUS mRNA stability to sustain shoot apical meristem (SAM) homeostasis; heat stress disturbs this module, triggering SAM arrest followed by recovery. In protein‑quality‑control pathways, the binding immunoglobulin protein 2 (BIP2)‑dependent unfolded protein response (UPR) is activated upon heat‑induced misfolded‑protein accumulation to restore protein function and prevent stem‑cell loss. For systemic signaling, S‑nitrosoglutathione (GSNO) acts as a long‑distance signal transported through plant vascular tissues to communicate heat‑stress signals between the SAM and root apical meristem (RAM).

The translational significance of this perspective is currently limited. Compared to the better-characterized HSF-HSP pathway, mechanisms associated with stem cell homeostasis have not yet been broadly validated in crop breeding practices. Their most immediate contribution may lie less in direct deployment than in broadening the conceptual landscape of plant thermotolerance and identifying developmental control points that can be incorporated into future crop improvement strategies, together with classical stress response pathways (Figure 4).

## 6. Improvement strategies for heat tolerance during the plant reproductive stage

Across plant developmental phases, the reproductive stage is generally the most vulnerable to heat stress. Elevated temperatures can sharply reduce seed set and final yield by disrupting pollen viability, stigma receptivity, pollination, fertilization, and grain filling. Therefore, enhancing heat tolerance at the reproductive stage has become a major focus of both crop breeding and heat stress research. Current approaches can be broadly grouped into genetic improvement, biotechnology-based precision improvement, and integrated regulatory or management strategies. These categories differ not only in terms of their technical basis but also in terms of their proximity to practical field deployment.^127^

### 6.1. Genetic improvement strategies

Heat tolerance is a complicated quantitative trait controlled by numerous genes and pathways; therefore, to improve thermotolerance during the reproductive stage, it is essential to first comprehend the genetic architecture of heat tolerance. The two main methods to identify loci associated with heat tolerance are genome-wide association studies (GWAS) and quantitative trait locus (QTL). QTL mapping in wheat has identified loci associated with traits such as stay-green, photosynthetic efficiency, and thylakoid membrane stability.^128–130^

Notably, TaHST2 is a known key locus controlling basal heat tolerance in allohexaploid wheat. It contributes to heat tolerance during both vegetative and reproductive stages and supports grain filling under heat stress, highlighting its potential value for wheat heat tolerance breeding.^131^ ^,^ ^132^ In soybean, the regulatory module composed of GmBSK1-GmGSK1-GmBES1.5 has potential value for breeding heat-tolerant cultivars.^133^ In rice, qPSLht4.1 and qHTB3-3 have been demonstrated to be associated with heat tolerance during the flowering period.^134^ In maize, loci related to grain set under heat stress have also been reported.^135^ ^,^ ^136^ In addition, *ZmHSF10* in maize plays a significant role in regulating plant heat tolerance and may provide useful genetic resources for enhancing heat tolerance in maize seedlings.^137^ Among these traits, stay-green has attracted particular attention in cereals because of its close association with both heat tolerance and yield during grain filling.^128^ ^,^ ^136^ ^,^ ^138^

Owing to its higher resolution and capacity to detect multiple allelic variants, GWAS has identified numerous candidate loci and genes associated with reproductive-stage heat tolerance in rice, maize, and wheat.^139–142^ The application of multi-parent populations, such as Nested Association Mapping and Multiparent Advanced Generation Inter-Cross, has enhanced the resolution of heat tolerance locus analysis.^143^ Therefore, genomic selection offers a means of capturing the combined effects of major and minor genes by integrating genome-wide marker information with phenotypic data, thereby substantially accelerating breeding for complex traits.^144–146^ Additional approaches, including speed breeding, doubled haploid breeding, and haplotype-based breeding, provide efficient routes for the rapid pyramiding of favorable heat tolerance alleles.^147–151^

From a translational standpoint, genetic improvement remains one of the most directly deployable strategies because it can be incorporated into existing breeding pipelines. However, a major limitation is that mapped loci remain strongly context-dependent and are influenced by genotype-by-environment interactions. Consequently, the practical value of QTLs and candidate loci ultimately depends on their validation across environments, developmental stages, and genetic backgrounds.

### 6.2. Biotechnology-driven precision improvement

Beyond conventional genetic improvement, modern biotechnology offers more targeted routes for optimizing heat tolerance traits during the reproductive stage. Transgenic strategies can rapidly enhance plant thermotolerance by introducing exogenous heat tolerance genes or by overexpressing endogenous regulators of heat responses.^152^ ^,^ ^153^ Candidate genes identified to date include HSFs, HSPs, antioxidant enzymes, and several classes of TFs.^154^ ^,^ ^155^ For example, in rice, the overexpression of OsHSP18.6 reduces heat-induced sterility.^156^ In soybean, reduced expression of Glycine max drought-induced SINA (*GmDIS1*) enhances both drought resistance and heat tolerance.^157^ In maize, ZmCDPK7 enhances heat tolerance by regulating the antioxidant system and HSP expression.^158^ ^,^ ^159^ In wheat, overexpression of *TaHsfA2H* can also significantly enhance heat tolerance by upregulating genes involved in ABA and ROS signaling pathways.^160^ Furthermore, the manipulation of key enzymes involved in GABA biosynthesis, starch metabolism, and phenylpropanoid metabolism has been shown to improve cereal adaptation to high temperatures.^161–165^

Genome editing technologies, particularly CRISPR-Cas9, combine precision with high efficiency and therefore have been recognized as robust tools to augment plant heat tolerance.^166^ ^,^ ^167^ These technologies also carry immense potential for application in gene function research and for utilization in breeding crop varieties that are resilient to multiple stress factors.^168^ These systems allow the modification of expression of genes with a key role in heat response pathways through gene knockout, targeted insertion, or site-specific mutagenesis, thereby avoiding some of the linkage drag commonly found in conventional transgenic approaches.^169^ Although the application of genome editing to reproductive-stage heat tolerance in cereals is still in its early stages, current studies indicate that the precise editing of major heat tolerance regulators can substantially improve thermotolerance.^170^

In contrast, biotechnology-driven precision improvement continues to face several practical constraints, including limited transformation efficiency, dependence on tissue culture systems, regulatory barriers, and potential trade-offs between stress tolerance and growth. Accordingly, although these approaches are highly valuable for functional validation and rational trait design, their effective incorporation into breeding programs will require stronger integration with field-based trait assessment and breeding-compatible delivery platforms.

### 6.3. Supporting regulatory and integrated management strategies

Beyond genetic and molecular improvements, the enhancement of heat tolerance during the reproductive stage also relies on the coordinated application of candidate gene discovery and agronomic management. Transcriptomic, proteomic, and metabolomic studies have provided robust support for the efficient identification of genes and pathways associated with heat tolerance.^171^ ^,^ ^172^ Comparisons between heat-tolerant and heat-sensitive genotypes under high-temperature conditions have revealed large sets of differentially expressed genes, proteins, and metabolites.^173^ For instance, members of the WRKY and ERF families have been proven to be associated with heat tolerance during rice panicle development.^174^ In maize, the suppression of genes associated with carbohydrate metabolism has been identified as an important cause of grain abortion following post-flowering heat stress.^175^ Proteomic and metabolomic analyzes have further shown that the accumulation of photosynthesis-related proteins, antioxidant proteins, HSPs, and diverse amino acid metabolites is closely associated with heat tolerance.^176–178^ These findings provide a basis for candidate gene screening and molecular breeding and are more useful as discovery resources than as directly deployable solutions.^179^

In field practice, agronomic regulation is an important complementary strategy for mitigating reproductive-stage heat damage. Adjusting sowing time can help crops avoid periods of high temperatures during flowering or grain filling, thereby reducing the risk of heat stress.^180^ ^,^ ^181^ Appropriate water and nutrient intake management can support physiological homeostasis and enhance tolerance to short-term heat exposure.^182–184^ The yield stability could be further enhanced by adopting protective cultivation practices aimed at conserving soil moisture and improving resource use efficiency.^180^ ^,^ ^181^ ^,^ ^185^ Heat pretreatment can also induce heat memory, thereby strengthening tolerance to the subsequent event of exposure to high temperature during the reproductive stage.^186–188^ Exogenously applied compounds, such as ABA, BRs, salicylic acid, melatonin, proline, betaine, and spermidine, can mitigate thermal damage by activating heat response pathways and antioxidant systems.^189–191^ The application of plant growth-promoting bacteria has been reported as a promising alternative to improve the heat tolerance of cereals during their reproductive stage.^192^ ^,^ ^193^

Overall, agronomic and integrated management approaches are currently the most immediately applicable options in the field, whereas gene-centered and omics-assisted strategies could provide optimal results over medium to long term. Future advances will probably depend on the integration of these technologies, rather than using them as separate entities.^194–197^

In addition to the above, recent advances in single-cell and spatial transcriptomics have overcome the limitations of traditional bulk transcriptomics, which fail to resolve cellular heterogeneity and spatial gene expression patterns.^198^ These technologies now provide high-resolution spatiotemporal molecular insights for understanding crop stress resistance mechanisms and enabling precision breeding. Based on the Breeding 5.0 intelligent breeding framework, AI technologies span the entire breeding pipeline-from predicting gene targets and assessing sgRNA risks to designing functional proteins and conducting high-throughput phenotyping-shifting breeding from empirical selection toward a data-driven, targeted design paradigm.^199^ In the future, integrating single-cell and spatial transcriptomic data, building interpretable AI models guided by biological mechanisms, and coupling gene editing with intelligent phenotyping tools will significantly enhance the efficiency of developing heat-tolerant crop germplasm, providing critical technological support for food security under climate warming.

## 7. Summary and future perspectives

As sessile organisms, plants exhibit intriguing interconnections between their development and responses to a variety of abiotic stresses, including osmotic stress, salinity, temperature extremes and drought. Notably, each type of abiotic stress activates distinct molecular signaling pathways tailored to its unique characteristics. Over the course of long-term evolution, plants have evolved diverse alterations to cope with each abiotic stress. Nevertheless, different abiotic stresses may trigger similar physiological and morphological changes in plants.

Heat stress is one of the major environmental constraints faced by plants, which affects their growth, development, and productivity. This review summarizes the effects of high temperatures on plants at the physiological, morphological, and molecular levels. It focuses on alterations in cell structure, signal transduction through second messengers, and protein dynamics, and discusses multi-level response networks involving epigenetic regulation, transcriptional regulation, post-transcriptional regulation, and thermal memory. Stem cell homeostasis is also highlighted as an emerging component of thermotolerance, and current strategies to enhance heat tolerance during the reproductive stage have been evaluated.

According to recent research, stress sensing, signal transduction, transcriptional reprogramming, proteostasis, and developmental control contribute synergistically to control plant thermotolerance. While the best-characterized core module is still the HSF-HSP pathway, accumulating data suggest that meristem maintenance, control at the RNA level, and chromatin regulation are also crucial to explain how plants maintain growth, recover from stress, and retain reproductive success especially under high temperatures. Nonetheless, there are significant gaps, particularly in our knowledge regarding the mechanism through which heat perception influences plant development and yield, the pathway that ensures the preservation of developmental stability under heat stress, and the approach for converting the gained mechanistic insights into strategies for improving crop productivity under actual field conditions.

The following three major areas should be the focus of future research. First, it should clarify how various heat-sensing events are incorporated into a coordinated regulatory framework that links initial cellular signaling with long-term impact on development and yield. Second, mechanisms that preserve developmental stability under heat stress should be thoroughly evaluated, especially stem cell homeostasis and reproductive-stage resilience, as these processes strongly influence sustained growth and final productivity. Third, in addition to mechanistic discoveries, researchers should focus on determining heat-resistant regulatory factors that remain effective under various genotypes, developmental stages, and complex environments, particularly under combined stress conditions such as heat stress and drought. Advancements in these aspects are critical to enable the development of a more complete framework of plant adaptation to high temperatures and to guide the breeding of climate-resilient crops.
